# **D**is**in**tegrate (DIN) Theory Enabling Precision Engineering of Proteins

**DOI:** 10.1021/acscentsci.2c01455

**Published:** 2023-01-23

**Authors:** Preeti Chauhan, Ragendu V., Mohan Kumar, Rajib Molla, Unnikrishnan V. B., Vishal Rai

**Affiliations:** Department of Chemistry, Indian Institute of Science Education and Research Bhopal, Bhauri, Madhya Pradesh 462066 INDIA

## Abstract

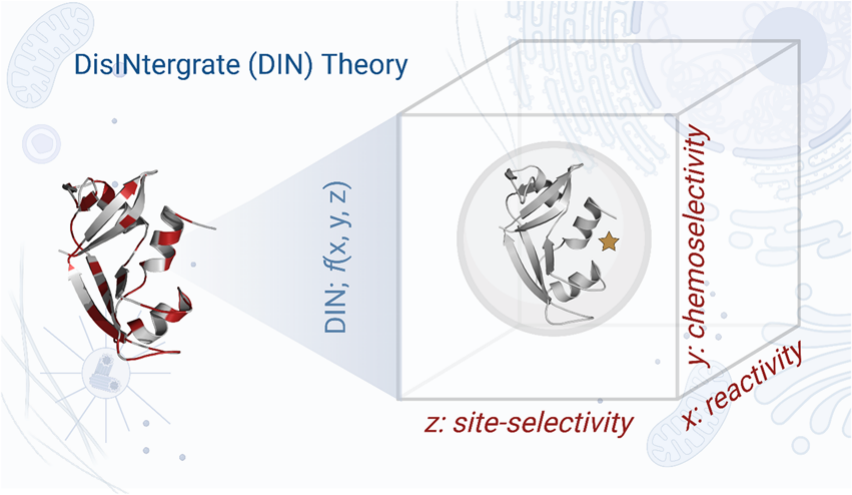

The chemical toolbox for the selective modification of proteins has witnessed immense interest in the past few years. The rapid growth of biologics and the need for precision therapeutics have fuelled this growth further. However, the broad spectrum of selectivity parameters creates a roadblock to the field’s growth. Additionally, bond formation and dissociation are significantly redefined during the translation from small molecules to proteins. Understanding these principles and developing theories to deconvolute the multidimensional attributes could accelerate the area. This outlook presents a **d**is**in**tegrate (DIN) theory for systematically **d**isintegrating the selectivity challenges through reversible chemical reactions. An irreversible step concludes the reaction sequence to render an **in**tegrated solution for precise protein bioconjugation. In this perspective, we highlight the key advancements, unsolved challenges, and potential opportunities.

## Abstract 2

The DIN theory provides a systematic tool to **d**isintegrate the selectivity challenges in protein bioconjugation, followed by the **in**tegration of their solutions using a combination of chemical reactions.

## Introduction

The synchronized coordination of events within biomolecules regulates human health. In this pool, proteins have served as valuable candidates as both therapeutics^1^ and targets.^2^ While the former requires labeling in isolated form, the latter depends on their selective targeting in a complex biological milieu. The screening-driven drug discovery processes remain the preferred routes for the latter.^3^ Further, proteome-wide chemoproteomics has accelerated the segment by establishing selective probes and new targets.^4,5^ On the other hand, the data also provide insight into considerable drug promiscuity and its implications.^6^ These developments highlight the need for principles to regulate selectivity in protein modification.

**Figure 1:**
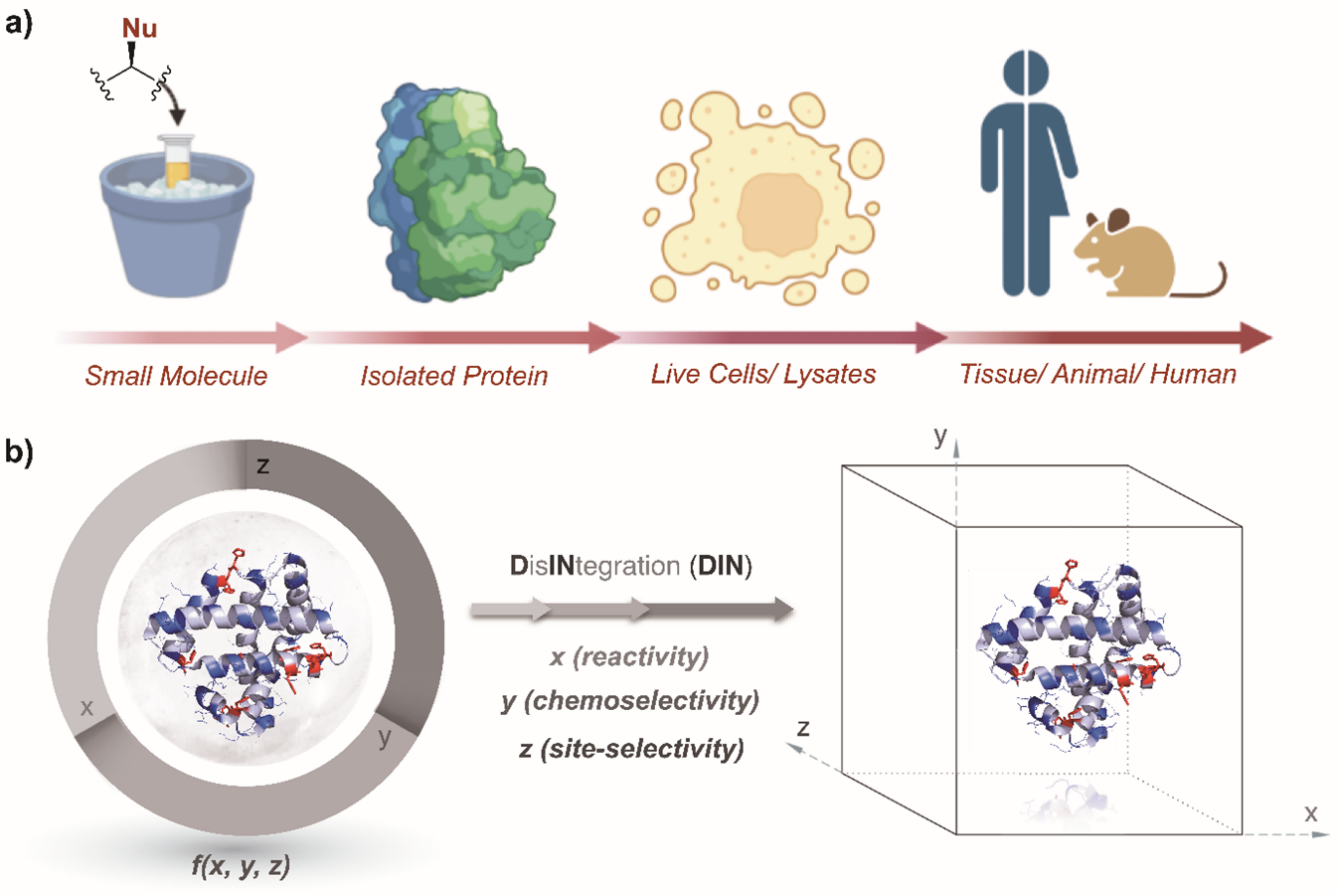
(a) The microenvironment of a functionality (nucleophile, Nu) from small molecules to a complex protein-based social ecosystem. (b) DIN theory: the **d**isintegration of reaction attributes and their independent regulation can empower the **in**tegration of solutions for the precision engineering of native proteins.

In this direction, we first need the tools and methods that can allow us to control selectivity with isolated proteins. Next, we can translate them sequentially from one system to another (Figure 1a). In the process, we can understand the impact of the complexity added by the microenvironment layers within cells, tissues, animals, and humans. This approach can complement the ongoing efforts to develop covalent regulators and inhibitors.^7−9^ Besides, controlling selectivity with isolated proteins, enzymes, and antibodies would empower biologics.^10−14^ The overlap of knowledge between segments at the two ends of the complexity scale, from small molecules to humans, must evolve with time to meet the technological demands.

Revisiting this problem from the end of small-molecule substrates provides another perspective. It initiates with chemical bond formation between two reactive partners, such as nucleophiles and electrophiles (Figure 1a). With a limited number of variables, the characteristics of such organic transformations are predictable. However, the behavior spectrum broadens once additional functional groups accompany the nucleophile. As a simple example, the bond-formation capabilities of a primary amine in *n*-butyl amine, a Lys-bearing peptide, and a Lys-bearing protein could be substantially different. The trend continues with additional layers added to the functional group ecosystem (Figure 1a). Can we have a theory to deconvolute the spectrum of a functional group’s behavior in a complex social environment? It could promote hypothesis-driven research and bring a larger protein landscape within the purview of selective modification. A chemical method for the precise single-site modification of an isolated protein needs to cross three critical barriers (Figure 1b). Initially, the reagent requires a reasonable reactivity at a low substrate concentration (*x*-axis). Next, the reagent must distinguish one functional group from others (chemoselectivity, *y*-axis). Finally, such a chemoselective reagent should be able to distinguish one copy of a residue from its multiple copies to display site selectivity (*z*-axis). As we move forward, the modularity beyond protein-defined reactivity order, site or residue specificity, and protein specificity can offer additional dimensions.

**Figure 2:**
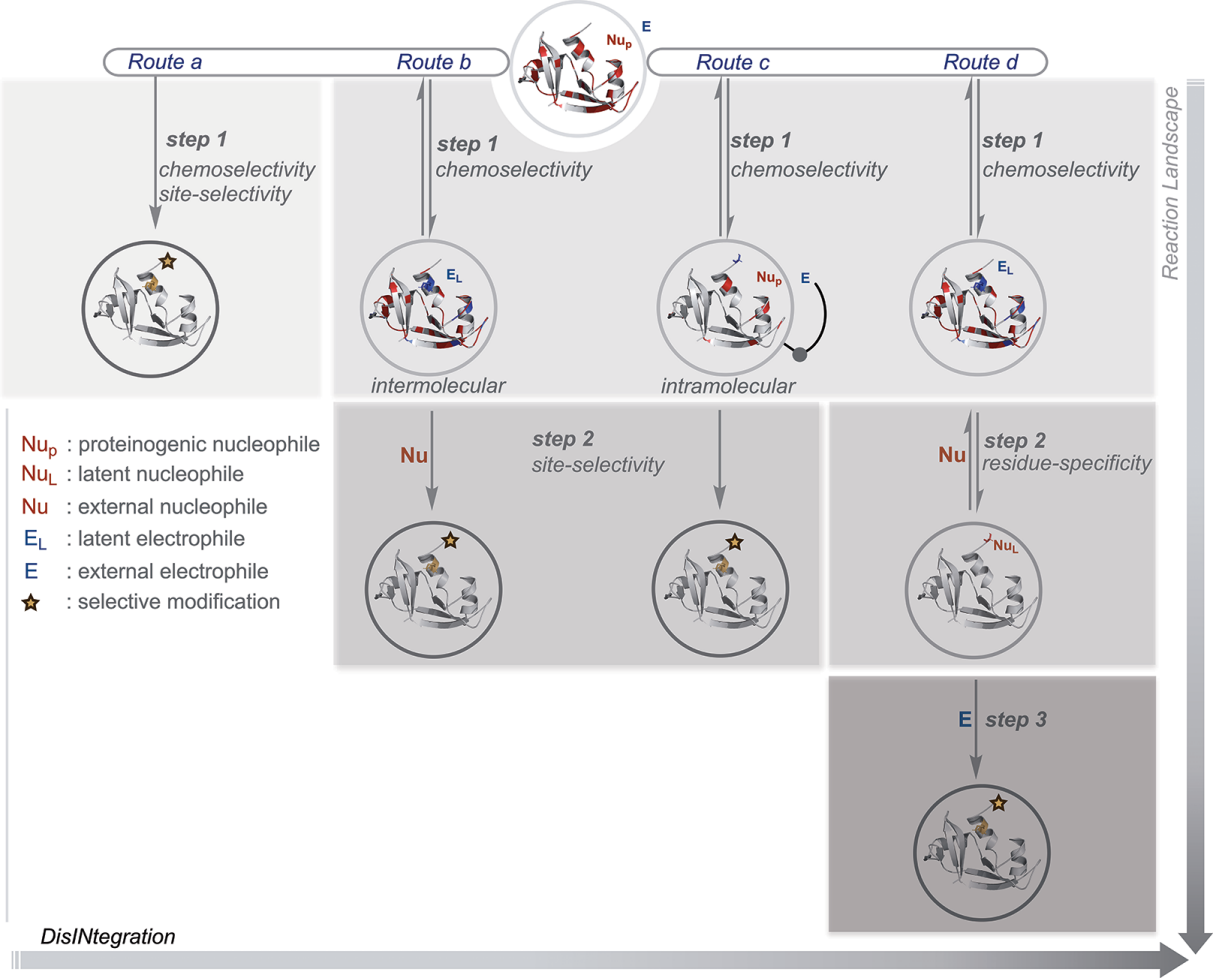
**D**is**in**tegration of selectivity attributes through chemical steps redefines the reactivity landscape to enable single-site protein modification. (a) Targeting reactivity hotspots in one step. (b) A two-step process, reversible intermolecular and irreversible intermolecular, can deconvolute chemoselectivity and site selectivity to render redefined reactivity hotspots. (c) A two-step process, reversible intermolecular and irreversible intramolecular, can offer modularity in addition to chemoselectivity and site selectivity deconvolution. (d) A multistep process can deconvolute chemoselectivity and residue specificity. The selected routes a–d show representative possibilities and can be extended to other reagent and intermediate combinations.

In this outlook, we argue that the selectivity attributes can be disintegrated through a multistep chemical transformation (DIN theory, Figure 2). Under such a case, the tuning of the selectivity attributes, such as chemoselectivity and site selectivity, can be done independently. Besides, such a deconvolution can create a redefined reactivity landscape of proteins to label residues beyond the hotspots, which are sites that offer the best combination of reactivity and solvent accessibility. In this perspective, Route A presents examples that address all the selectivity challenges in a single step (Figure 2).^15,16^ Subsequently, Route B outlines how chemoselectivity and site selectivity can be disintegrated into two steps. In the process, it redefines the reactivity landscape and hotspots. Further, Route C demonstrates how such multistep deconvolutions can offer a modular platform. Finally, we strengthen the argument through Route D, which demonstrates the segregation of chemoselectivity and residue-specificity for single-site protein bioconjugation. While the general sections are directed toward a broad readership, the exemplification would benefit the researchers in the field. Overall, it demonstrates how DIN theory could empower hypothesis-driven research to find new selectivity attributes and target unique sites/domains that have not been accessible to precision protein engineering technologies.

## Route A

The initial efforts for the precision labeling of proteins involved addressing all the selectivity attributes in a single step (route a, Figure 2). Typically, such transformations would apply an irreversible chemical transformation. However, there are examples where a reversible step regulates both chemoselectivity and site selectivity, while an irreversible transformation would render the product. In both cases, proteinogenic residues’ inherent reactivity order and solvent accessibility determine the modification site. Consequently, such methods offer a narrow range of reaction parameters under which absolute single-site selectivity can be achieved. Such methods engage only a single residue for its irreversible transformation in the overall process. Additionally, the bioconjugation reagent’s size, structure, and binding preferences could also contribute. On the other hand, the protein’s structure plays a defining role in establishing the reactivity hotspots. Its perturbation provides a gateway to access the low-frequency residues with limited solvent accessibility. Although, the same would compromise the site selectivity for high-frequency residues. The protein bioconjugation methods in this Outlook were primarily developed under nondenaturing physiological conditions unless specified.^17,18^

**Figure 3:**
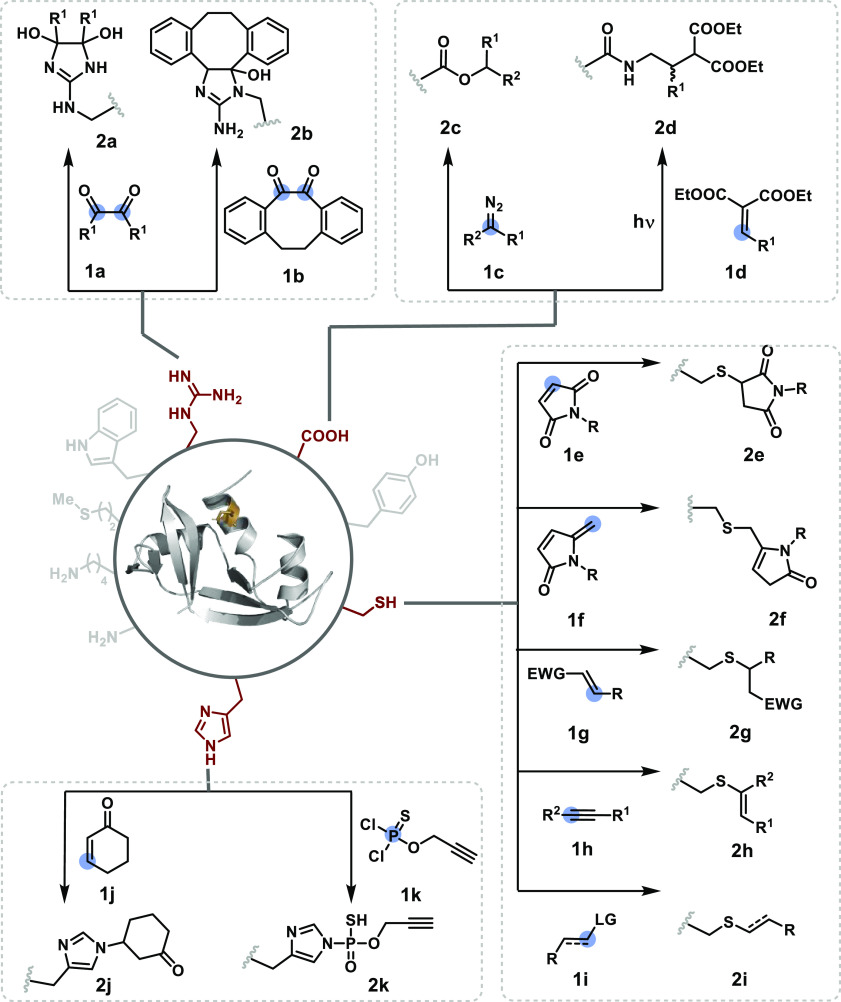
Targeting reactivity hotspots in one step: efforts with Arg, carboxylates, Cys, and His.

The nucleophilic functionalities in a protein are also basic in nature. Their p*K*_a_ values determine the concentration of their deprotonated or nucleophilic form in the reaction mixture. In turn, it contributes to their reactivity order. For example, Arg is largely protonated under physiological conditions. Its reaction with diketones (**1a**, Figure 3), e.g., cyclohexanedione, typically requires alkaline conditions.^19^ The side-chain guanidine and cyclohexanedione result in irreversible Arg modifications. However, the elevated pH also activates multiple Lys and His residues. Hence, the Arg modifications are often accompanied by chemoselectivity challenges. The dibenzocyclooctendione (**1b**) motif creates an opportunity for irreversible benzylic rearrangement after the nucleophilic addition of Arg.^20^ The interplay of reversible and irreversible pathways adds another dimension to chemoselectivity. For example, oxoaldehyde reacts with Lys in a kinetically preferred transformation. However, Arg delivers a thermodynamically stable product, while the Lys adduct reverts through transoximization with hydroxylamine.^21^

Unlike Arg, carboxylic acid remains in deprotonated carboxylate form under physiological conditions. However, its low nucleophilicity makes it very difficult to target with chemoselectivity. Besides, its high abundance creates a roadblock to site selectivity. For example, esterification with diazo compounds (**1c**) can lead to the labeling of multiple carboxylates without distinguishing Asp, Glu, and C-terminus CO_2_H (**2c**).^22,23^ On the other hand, the oxidation potential differences between C-terminal and side-chain carboxylates enabled site-selective C-terminal radical generation (**2d**).^24^ An α,β-unsaturated ester traps the relatively high-energy C-radical to render the bioconjugate. Even though the oxidative conditions or electrophilic system can compromise the extent of conjugation, it is a positive step toward the selective targeting of a residue with low reactivity.

Cysteine offers the other end with respect to carboxylates, both in terms of occurrence and reactivity. Its low frequency often means that the site selectivity is not applicable for achieving single-site protein bioconjugation. On the other hand, chemoselectivity can be achieved with a range of soft electrophiles. Multiple polarized double bonds have been identified from this perspective. For example, the conjugate addition using maleimide derivatives has been explored extensively for Cys modification (**1e**).^25−27^ However, the inherent reversibility of C–S bonds results in thiol exchange and could compromise the chemoselectivity and site selectivity. These challenges have been addressed by regulating the reactivity through subtle changes in the electrophile. For example, incorporating bromine,^25^ an exocyclic double bond,^28^ and the hydrolysis of the product^29^ has offered bioconjugates with higher stability. If an exocyclic double bond replaces the maleimide carbonyl, the 1,4-addition and subsequent reduction yield the bioconjugate over pH 6.0–8.5 (**1f**).^30,31^ However, such methylene pyrrolones are susceptible to retro-Michael addition and thiol exchange under basic conditions. In another set of electrophiles, the carbonyl acrylates^32^ offer better stability than azidoacrylates.^33^ Additionally, the vinylpyridinium salts offer irreversible Cys modification with negligible competition from Lys.^34^ Besides, the isoxazolinium ring uses intramolecular rearrangement and fragment release to render a stable and site-selective Cys adduct.^35^ The polarized double bonds equipped with electron-withdrawing groups also offer p*K*_a_ regulation to avoid retro-Michael addition, such as vinyl sulphone derivatives (**1g**).^36^ Polarized triple bonds, such as alkynoic amides, esters, and alkynones, offer another possibility (**1h**).^37^ In particular, they are preferred when the application needs cleavable reagents. An unsaturated vinyl sulfide linkage is created in such cases that undergoes an addition–elimination sequence in the presence of an external thiol. The positions of the alkyne, terminal or internal, and the electron-withdrawing functionality regulate their reactivity. For example, an internal alkyne polarized between aryl and cyano groups offers hydrolytically stable reagents.^38^ The alkylation reactions offer another opportunity for chemoselective Cys bioconjugation (**1i**). The nucleophilic substitution of halogens such as bromine (bromooxetane)^39^ or chlorine (chlorofluoroacetamides)^40^ occurs under mild conditions. However, a few bromooxetane derivatives require higher pH (8–11) and an organic solvent. The nucleophilic aromatic substitution with benzothiazole^41^ or chlorotetrazine^42^ also renders Cys selectivity, with some challenges from Lys. In a mechanistically different route, a free radical-mediated pathway can render thio-ene-^43^ and thio-yne-based^44^ Cys modifications. The former involves the photoinduced coupling of alkenyl glycosides with the thiol group. On the other hand, thio-yne coupling proceeds by the addition of two thiol-based free radicals to a terminal alkyne. The vinyl sulfide intermediate formed by the addition of the first thiol is captured by a second thiol via a thio-ene mechanism. However, both methods also engage other residues, including disulfide bonds, and impact the structural integrity of the protein.

The reversibility becomes even more prominent with the His side chain due to the inherently labile imidazole-based N–C bond. This moderate frequency residue with low reactivity poses a stern challenge for single-site modification. Substantial competition from Cys and Lys is unavoidable in most cases. Despite this, the prominent role of His in biological pathways makes it a valuable target.^45^ The reversibility of the His-based Michael adduct can be reduced by altering the p*K*_a_ of the β-proton through a functional group transformation.^46^ However, such an approach does not address the chemoselectivity. Interestingly, cyclohexenone (**2j**)^47^ and thiophosphorodichloridates (**2k**)^48^ offer noteworthy control over chemoselectivity and site selectivity for single-site His modification.

**Figure 4:**
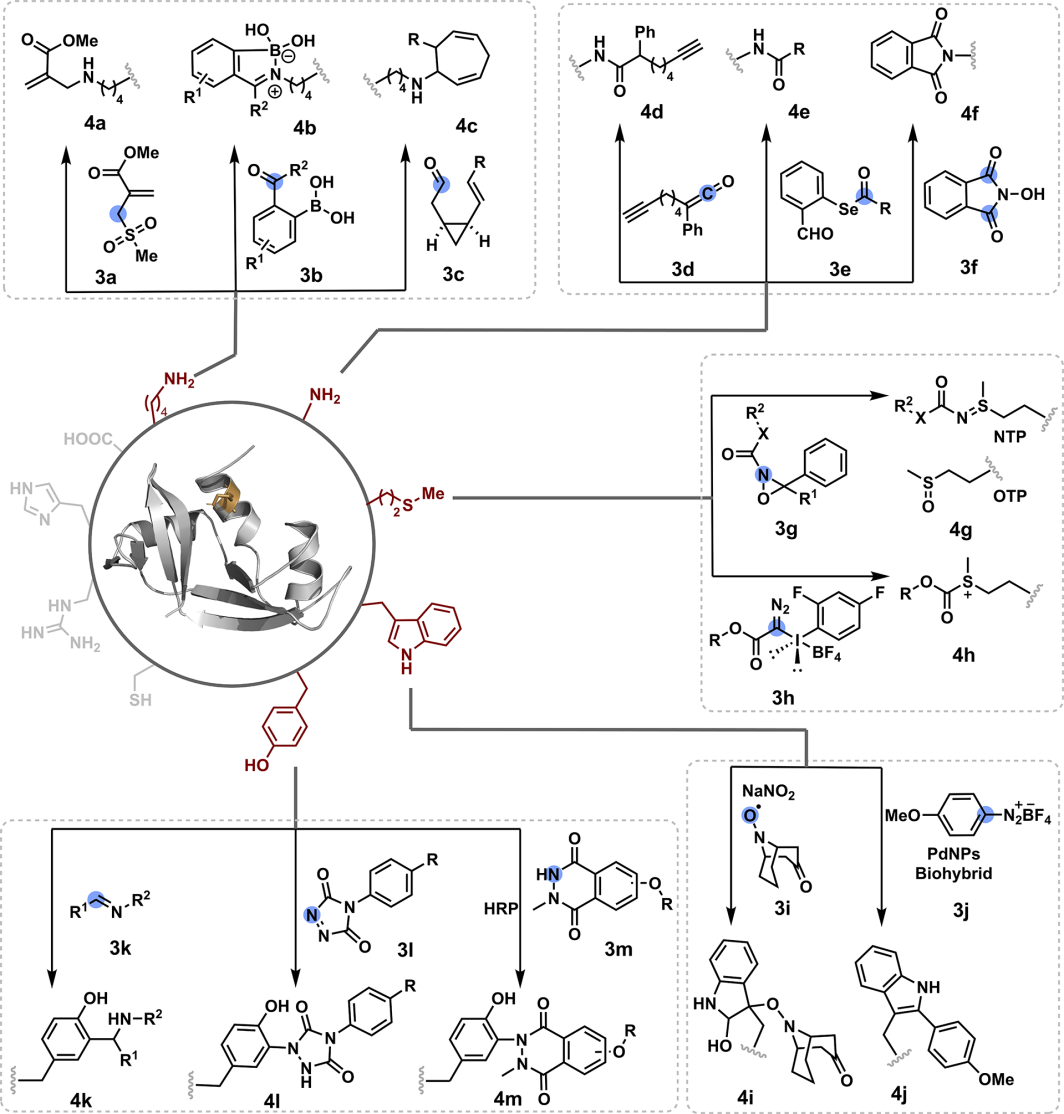
Targeting reactivity hotspots in one step: efforts with Lys, N-terminus amine, Met, Trp, and Tyr.

Unlike His, multiple electrophiles have been established to display high chemoselectivity toward the N-terminus α-amine (*N*^α^-NH_2_) and the Lys ε-amine (*N*^ε^-NH_2_). Cys is a prominent competitor for amines. However, the differences in hardness, p*K*_a_, and occurrence frequency enable their exclusive bioconjugation. The p*K*_a_ difference also makes *N*^α^-NH_2_ a preferred target over *N*^ε^-NH_2_ under physiological conditions. The charged state ensures enhanced solvent accessibility for this high-frequency residue, making site selectivity a daunting task. Amine modification is often dominated by addition and substitution reactions. Amine acylation with NHS ester derivatives is one of the most frequently employed methods.^49^ The stability of an amide bond compared to that of a Cys-based thioester enables the formation of chemoselective product. However, such methods cannot distinguish between *N*^α^-NH_2_ and *N*^ε^-NH_2_. pH fine-tuning or the controlled addition of an NHS ester could deliver a site-selective *N*^α^-NH_2_ modification.^50^ The other C-centered amine-selective electrophiles include dichlorotriazine,^51^ sulfonyl acrylate (**3a**, Figure 4),^52^ and allyl isothiocyanate.^53^ The reversibly formed adducts with amine have also been exploited through subsequent stabilization or an irreversible reaction for chemoselective bioconjugation. For example, imines from a protein were trapped as iminoboronates (**3b**).^54^ In another case, the generated imine was designed for an irreversible [3 + 3] cycloaddition (**3c**).^55^ The *o*-ester-substituted arenediazonium reacts with the amine to generate an unstable triazine adduct. Next, an intramolecular reaction generates a stable benzotriazinone derivative.^56^ The site selectivity presents a substantial challenge in most of these cases. However, blocking *N*^ε^-NH_2_ through acidic reaction conditions could create an opportunity for N-terminus labeling. For example, this approach enabled a ketene to render single-site *N*^α^-NH_2_ labeling (**3d**).^57^ In a distinct approach, a hemiaminal formed between *N*^α^-NH_2_ and aryl aldehyde with *ortho*-selenoester enables the N-terminus modification (**3e**).^58^ Further, we demonstrated that a proximally placed electrophile could site-selectively capture the *N*^α^-NH_2_ imine.^59^ In another example, *N*-hydroxypthalimide achieves the site-selective *N*^α^-NH_2_ modification by shifting the rate-determining step from an intermolecular to intramolecular process while negating the requirement of slow addition (**3f**).^60^ The site-selective Lys modification is not accessible through single-step processes, especially due to the presence of *N*^α^-NH_2_.

Met presents an entirely different scenario with respect to Lys. It is among the most hydrophobic residues and hence is often buried in domains with minimal solvent accessibility. Moreover, it holds the second position from the bottom in the amino acid frequency scale. These attributes make surface-accessible Met residues rare. It is less likely to face site selectivity challenges when available, making it a promising target for single-site modification. As it is prone to oxidation, redox-activated chemical tagging (ReACT) enables selective Met bioconjugation using oxaziridine-based reagents (**3g**).^61,62^ Hypervalent iodine reagents also offer promise from this perspective (**3h**).^63^ However, selectively modifying Met under mild conditions without perturbing other residues is still problematic.

Principally, Trp falls under a similar category as Met, as it is the least abundant and exhibits minimal surface exposure. Hence, it provides an equally good opportunity for single-site protein bioconjugation. A sterically unhindered and stable nitroxyl radical (ABNO) reacted selectively with Trp by forming a C(3)–O bond with indole (**3i**).^64^ In another case, a heterogeneous PdNP biohybrid catalyst allowed indole C(2)–H activation in Trp under physiological conditions (**3j**).^65^ The protein (Cal-B) offers two Trp residues on the surface, and careful control of catalyst loading allows the predominant labeling of a single site.

Tyr is the third residue in this category, with a low frequency and limited surface exposure. The C=N and N=N bonds in diverse structural motifs have served as capable electrophilic systems for targeting the phenolic residue of Tyr. For example, Tyr reacts conveniently with an in situ formed imine^66^ and cyclic imines (**3k**).^67^ Besides, the cyclic diazodicarboxamide derivatives have been successfully employed for selective Tyr bioconjugation through the ene-reaction under physiological conditions (**3l**).^68,69^ Additionally, a diazonium salt offers an appropriate handle to react with Tyr chemoselectively.^70^ In another approach, *N*-methyl luminol derivatives under HRP-catalyzed single-electron transfer (SET) or electrochemically activated SET deliver selective Tyr modification (**3m**).^71,72^

### Ligand-Directed Modification

**Figure 5:**
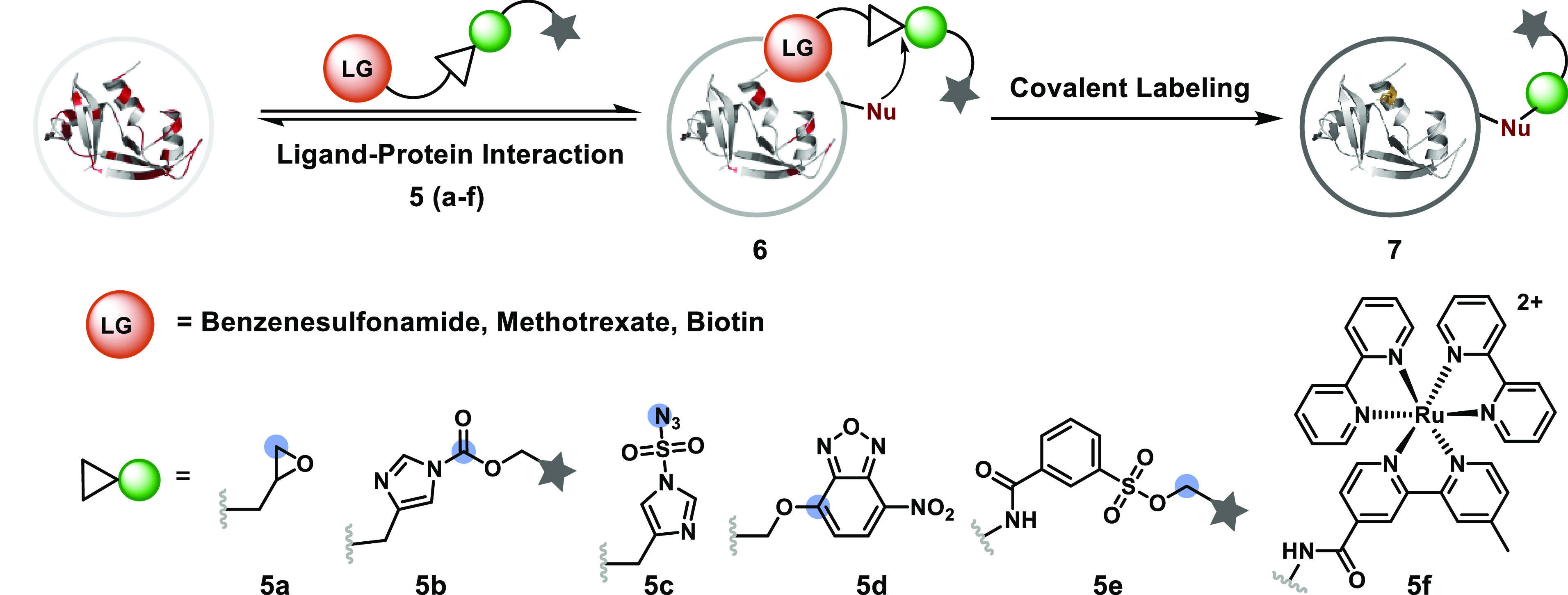
Ligand-enabled localization reduces competitors and empowers the single step for the selective labeling of proteins.

The challenges of addressing reactivity, chemoselectivity, and site selectivity are amplified with large proteins and complex biological mixtures. The ligand–protein interaction driven by reversible covalent or noncovalent binding offers an excellent solution by limiting the number of competitors. Subsequently, the electrophile has a much better opportunity to render a chemoselective and site-selective modification. In this perspective, a benzenesulfonamide ligand tethered to an epoxide (**5a**, Figure 5) through a linker could deliver the selective labeling of His in human carbonic anhydrase II (hCAII).^73^ The other initial findings established that varying the linker alters the reactivity and site selectivity of the epoxide in hCAII.^74,75^ This method offered the additional flexibility of removing the ligand post-bioconjugation. A benzenesulfonamide-linked tosyl group (**5e**) also offered similar control over selectivity, enabling His alkylation in hCAII.^76^ The ligand-enabled localization of acyl imidazole (**5b**) created an opportunity for the selective acylation of a single Lys residue in a protein.^77^ The same ligand was also utilized to localize a Ru complex ([Ru(bpy)_3_]^2+^, **5f**), allowing SET photocatalysis for selective Tyr modification.^78^ The concept has been further extended to the biotin–avidin combination for the selective labeling of Lys using *O*-nitrobenzoxadiazole (*O*-NBD, **5d**)^79^ and the diazotransfer reaction with imidazole-1-sulfonyl azide (**5c**).^80^ The specificity of the ligand–protein interaction limits the competitors considerably and empowers route a for selective protein modification in a biological milieu such as live cells.

## Route B

The DIN theory deconvolutes the chemoselectivity and site selectivity into two steps. In turn, it could provide enhanced control while addressing these selectivity attributes separately (route b, Figure 2). For example, a reversible first step can render chemoselectivity. In this case, the subsequent irreversible *intermolecular* reaction will only need to regulate the site selectivity. Besides, the intermediate generated after the first step could redefine the reactivity order. In turn, it opens the opportunity for single-site labeling of reactivity hotspots distinct from route a targets. Such methods could also offer more flexibility with the reaction parameters, at least until the first step.

**Figure 6:**
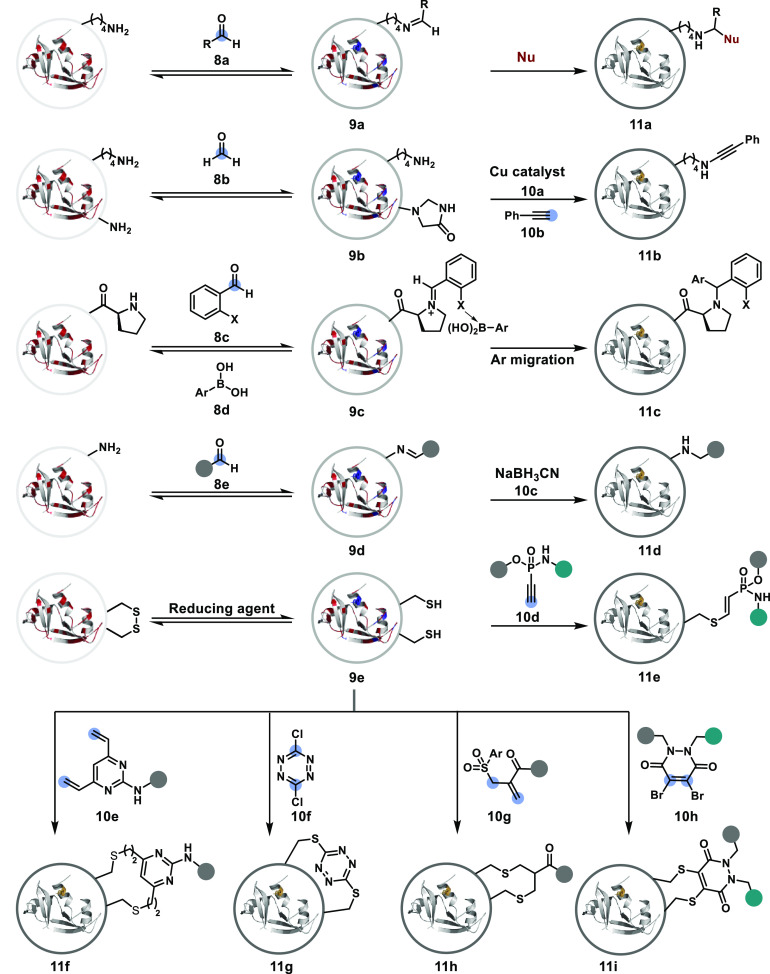
Route B: a two-step process can deconvolute chemoselectivity and site selectivity to render redefined reactivity hotspots.

In one of the established examples, the reaction of an aldehyde (**8a**, Figure 6) derivative with a protein reversibly and chemoselectively creates imine (**9a**). This step engages multiple solvent-accessible amines. The external nucleophiles can site-selectively capture a single copy of these intermediates. We demonstrated that diethyl phosphite, triethyl phosphite, and *t*-butyl isocyanide (Nu) could deliver the single-site labeling of Lys residues with a structurally diverse set of proteins (**11a**).^81^ The reactivity order of primary amines is mostly redefined when they are converted from their nucleophilic form to imine-based intermediates. Often, this leads to a distinct bioconjugation site. The selection of *o*-phthalaldehyde can also render stable adducts with external amines.^82^ However, it is accompanied by competing pathways that create roadblocks for chemoselectivity and site selectivity. On the other hand, the latent electrophilic imine-based intermediates (**9b**) can be captured site-selectively by the Cu–acetylide complex (**11b**).^83^ Interestingly, the *N*^α^-NH_2_-based imine with selected aldehydes reacts rapidly with the proximal amide bond to produce imidazolidinone (**9b**). This reversible in situ protection keeps *N*^α^-NH_2_ out of the competition. Besides, this approach could create conjugation sites distinct from those in route a, confirming the alteration of reactivity hotspots. These methods translate well to large proteins such as monoclonal antibodies (mAbs). The approach can also facilitate targeting a proteinogenic secondary amine with high selectivity. For example, an *N*-Pro-derived iminium intermediate (**9c**) reacts with an external nucleophile conveniently in a borono-Mannich reaction (**11c**).^84^ The *N*^α^-NH_2_-imine (**9d**) with certain aldehydes (**8e**) can also be captured with NaBH_3_CN (**10c**).^85^ However, *N*-Cys-containing proteins render a mixture of reductive alkylation and thiazolidine-based protein conjugates.

In the absence of an accessible free Cys residue, a chemoselective reversible reduction of the disulfide bridge provides a notable alternative. For example, ethynylphosphonamidates (**10d**) capture the thiolate for antibody modification.^86^ Further, the reagents with two electrophilic centers add value by rebridging. From this perspective, the reduction (**9e**) followed by the thiol-selective reaction with divinylpyridine (**10e**) works well to render the bioconjugate (**11f**).^87^ In a principally similar manner, *s*-tetrazines (**10f**),^88^ allyl or aryl sulfones (**10g**),^89−91^ and pyridazinedinones (**10h**)^92^ render the selective targeting of disulfide bonds. This approach requires caution, as the functionality often contributes to structural stability. For example, disulfide scrambling and heterogeneity are commonly observed with the antibodies.

This disintegration route can also be translated to radical-based intermediates. The C_4_-alkyl-1,4-dihydroxypyridine reagents can promote photocatalyzed chemoselective His-based radical cation generation.^93^ In turn, this could deliver an alkylated imidazole residue in the product. In another case, the chemoselective generation of the radical cation with Trp was demonstrated in the presence of *N*-carbamoylpyridinium salt and UV–B light. The N-centered radical recombines to form a Trp-labeled bioconjugate.^94^ The site selectivity is not accessible with these high-energy reactive intermediates in general.

## Route C

The deconvolution of chemoselectivity and site selectivity into two steps can also empower a method with additional controls (route c, Figure 2). For example, the reversible and chemoselective first step can be followed by an irreversible *intramolecular* reaction. Contrary to route b, a pair of proteinogenic residues will regulate the bioconjugation in such a case. Hence, the proximal control could bypass the inherent reactivity order and determine the conjugation site. Such a site-selective method can offer modular single-site protein bioconjugation. Additionally, it creates an opportunity to explore whether two or more residues can create unique signatures in a protein. Since the irreversible final step is intramolecular, such an approach offers a substantial kinetic advantage over the background intermolecular reactions. Hence, this route promises enhanced flexibility with the reaction parameters without compromising the selectivity attributes.

**Figure 7:**
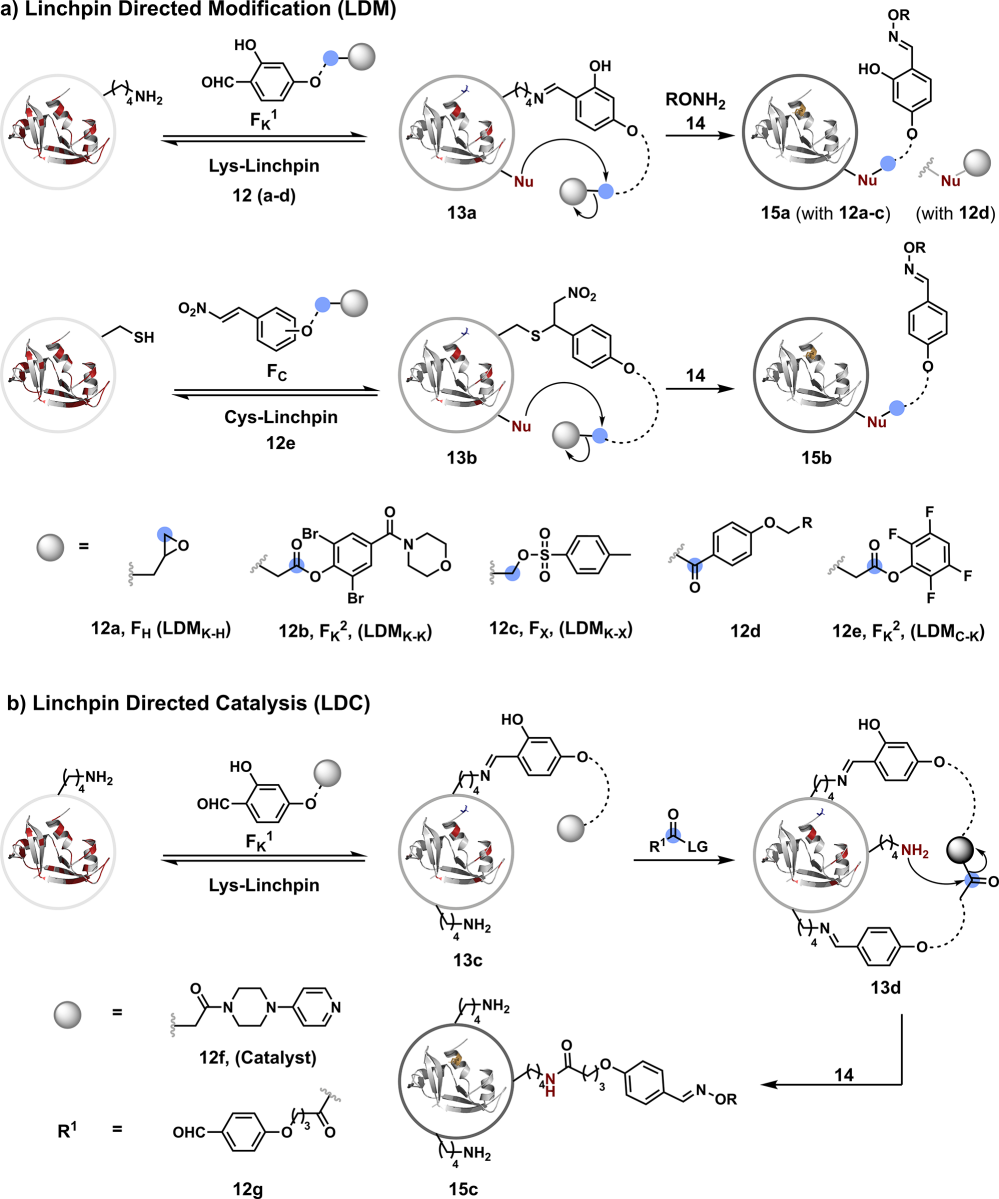
Route C: a two-step process can offer modularity in addition to chemoselectivity and site selectivity deconvolution.

The linchpin-directed modification (LDM) platform provides the proof of concept for this segment. Like route b, it initiates with chemoselective imine formation with all the accessible amines. However, this is where the similarity ends, as the imine does not participate in the subsequent irreversible reaction. It serves as a linchpin and directs another functional group to the proximal residue of interest. The chemoselectivity attributes of the latter and the spacer’s design connecting the two functionalities determine the conjugation site. For example, the *o*-hydroxybenzaldehyde (F_K_^1^, Figure 7a) tethered to an epoxide (**12a**, F_H_) through a linker gives a single-site His modification.^95^ The method offers simultaneous control over reactivity, chemoselectivity, site selectivity, and modularity. At first, F_K_^1^ forms an imine (linchpin, **13a**) with all the accessible Lys residues in a reversible reaction. It allows the second electrophile (F_H_) to react irreversibly with a proximal His residue to deliver site-selective labeling (**15a**). The site selectivity and modularity can be regulated by the spacer design. The aldehyde (F_K_^1^) is captured with hydroxylamine (**14**) for the subsequent installation of probes. The approach also translates well to the lysine-directed single-site modification of a lysine residue (LDM_K–K_) by replacing the epoxide (F_H_) with an acylating reagent (**12b**, F_K_^2^).^96^ The strategy was further extended to selectively label Lys residues in various therapeutically relevant monoclonal antibodies using *p*-phenol ester (**12d**) equipped with a linchpin fragment as the leaving group.^97,98^ The *o*-hydroxyl group of the linchpin imine makes it highly inert toward the external nucleophiles. It creates an opportunity to use another aromatic aldehyde (F_K_^2^) to create the second imine,^99^ which can be captured in the presence of an external nucleophile to deliver a single-site modification. The LDM platform also extends the site-selective modification of His or Asp using an alkylating reagent such as sulfonate esters (**12c**, F_X_).^100^ Additionally, we demonstrated that nitroolefins could shift the linchpin sites from a high-frequency residue (Lys) to Cys with low occurrence.^101^ This considerably reduces the number of competitors and creates the opportunity to translate the method for protein selectivity in a complex biological milieu. The nitroolefin (F_C_) meets all the criteria necessary to create an appropriate linchpin and use F_K_^2^ (**12e**) for the Cys-directed Lys modification (LDM_C–K_). Here, the chemoselective thio-Michael addition (**13b**) enables the site-selective Lys acylation (**15b**). The linchpin’s release from the thio-Michael adduct is coupled with a retro-Henry reaction to render an aldehyde under mild conditions for downstream utility. We anticipated that the geometry and conformation of LDM_C–K_ reagents would regulate the site selectivity. Hence, the protein modification must happen if the distance between the linchpin and the target site matches the F_C_–F_K_ effective length. Further, the contribution from the dynamics of the protein and the reagent needs to be assessed. In this perspective, the MD simulations offer microscopic insights into the structures and dynamics of these reaction partners. The simulation results validate that the conformational flexibility of the reagent coupled with protein-induced rigidity regulates the effective spacer length and the bioconjugation site. In another validation, the semioxamide vinylogous thioester-based STEF probes offer reversible Cys conjugation to regulate an irreversible site-selective Lys modification.^102^ A class of cleavable aryl thioethers linked with UV-activatable *o*-nitrobenzyl alcohol delivers the selective labeling of the Lys residue.^103^ The modified Cys residue is recovered by thiol exchange in this case. In a recent development, we established that this approach could be extended from a pair of residues to the molecular signatures composed of three residues (Figure 7b).^104^ It required disintegrating the acylating reagent into two components, the catalyst (**12f**) and the proelectrophile (**12g**), both of which were equipped with a linchpin handle for proximity control (LDC). In a principally similar approach, the reversible complexation of boronic acids (BA) with an antibody’s F_C_-*N*-glycan directs the 4-(dimethylamino)pyridine (DMAP).^105^ The latter forms *N*-acylpyridinium intermediate with thioester-based acyl donors to yield the acylation of a proximal Lys residue. Encouraged by the linchpin-guided protein modification platform, 10*H*-phenoxazine-3,7-dicarboxaldehyde was designed for Tyr modification via photoredox catalysis.^106^ It would be interesting to see if integrating spacers or linkers in these reagents can add modularity to this chemo- and site-selective method.

**Figure 8:**
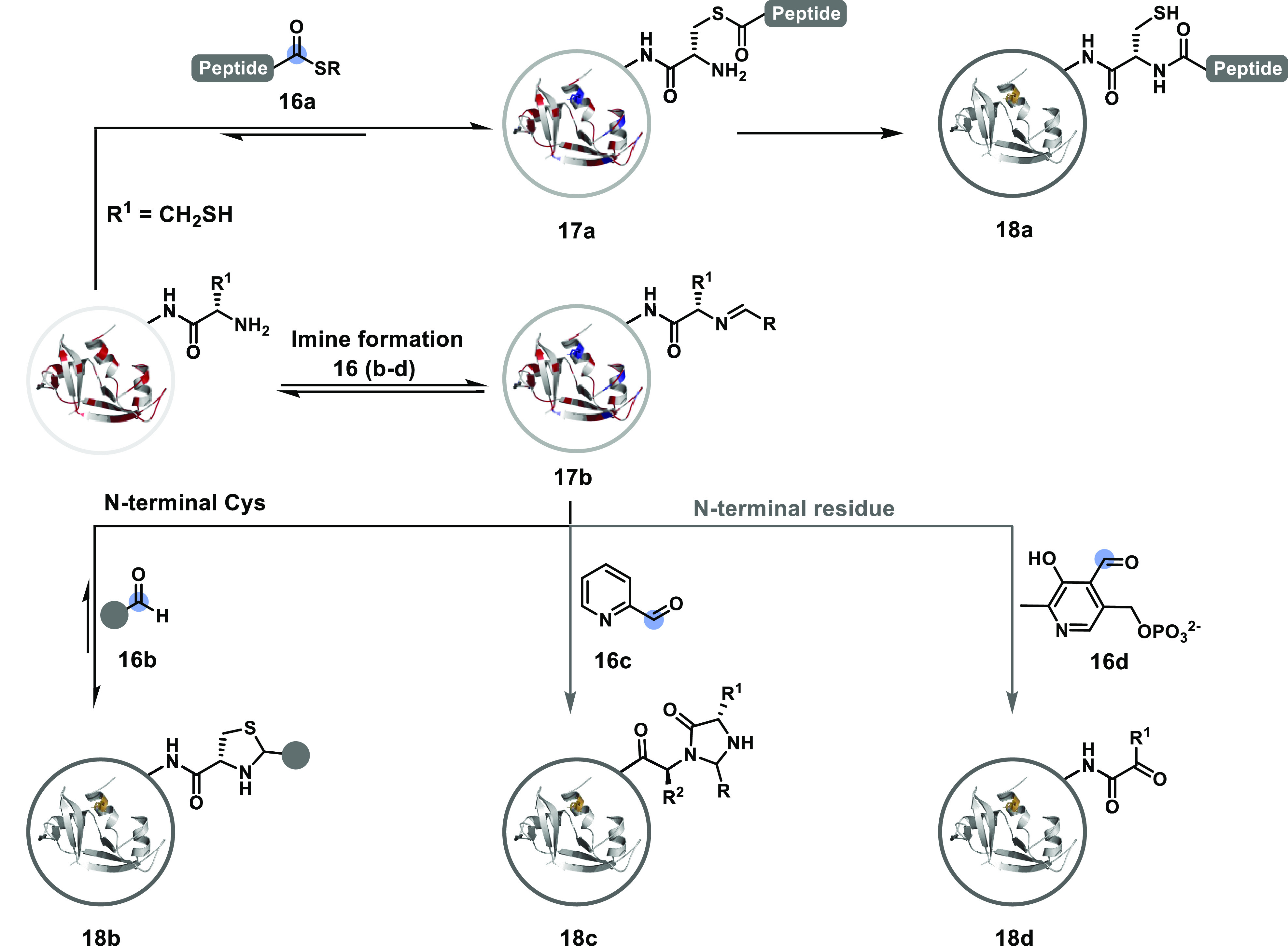
Route C: a two-step process can offer N-terminal specific modification.

Additionally, targeting a pair of functionalities from the same residue can offer a unique signature to drive selectivity (Figure 8). *N*^α^-NH_2_ coupled with another functional group, such as the side chain residue of the N-terminal amino acid, offers such an opportunity. For example, the *N*-Cys protein reacts reversibly with thioesters (**16a** to **17a**) to create an opportunity for an irreversible intramolecular S_Cys_ to *N*^α^-NH_2_ acyl transfer (native chemical ligation, NCL, **18a**).^107−109^ In another case, the *N*-Cys thiol could capture the *N*^α^-NH_2_-imine (**17b**) to render thiazolidine (**18b**).^110^ The challenges outlined for C–S bond stability in route a are associated with these adducts. However, they can be addressed to an extent through the use of aldehydes equipped with boronic acids.^111^ Boronic acid enhances the reaction rate and stabilizes the thiazolidine through the B–N dative bond along with other B-mediated coordination.^112^ The product stability can also be enhanced by engaging the lone pair on the thiazolidine nitrogen through intramolecular acylation.^113^ Additionally, the *N*^α^-NH_2_ can pair with the proximal amide to render selectivity. For example, 2-pyridinecarboxaldehyde (2-PCA, **16c**)^114^ or 1*H*-1,2,3-triazole-4-carbaldehyde (TA4C)^115^ forms an imine that prefers to react with the penultimate amide to yield imidazolidinone (**18c**). The Lys *N*^ε^-NH_2_-based imine lacks such support to facilitate the irreversible step. Under similar conditions, pyridoxal-5-phosphate (PLP, **16d**) generates the *N*^α^-NH_2_-imine that tautomerizes to render a glyoxyl imine.^116^ Its hydrolysis yields an aldehyde or ketone (**18d**) for chemically orthogonal transformations.

## Route D

**Figure 9:**
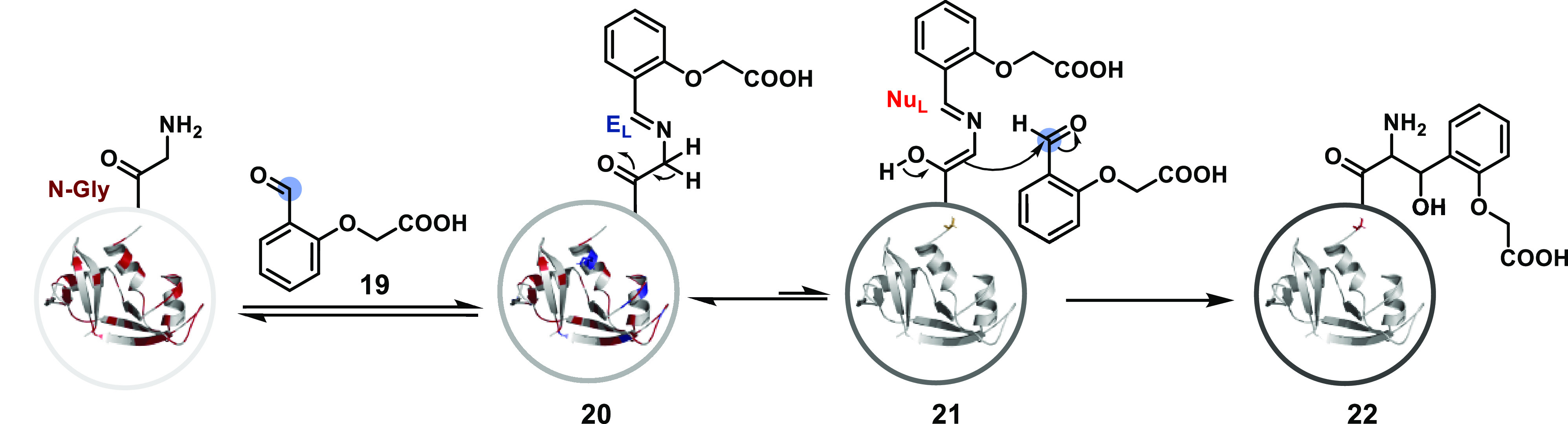
Route D: a multistep process can deconvolute chemoselectivity and residue specificity.

As we noticed in route c, treating the protein with an aldehyde chemoselectively converts a nucleophilic landscape to its electrophilic counterpart. If an additional step can follow this to generate a distinct reactive intermediate, it could disintegrate additional selectivity challenges. For example, we demonstrated that the *N*^α^-NH_2_-imine (**20**, Figure 9) with an aromatic aldehyde (**19**) could generate a nucleophilic intermediate (**21**).^117^ A proximal H-bond stabilizer in the aldehyde plays a critical role in the process. It allows the N-terminus *C*^α^-H functionality to be paired with *N*^α^-NH_2_ and creates a unique signature for bioconjugation. Hence, the *N*^ε^-NH_2_-imine would be excluded from any irreversible transformation. Additionally, the method can distinguish the *C*^α^-H of an unsubstituted amino acid from those of all the substituted analogs. Hence, it offers exclusive *N*-Gly selectivity and renders precision labeling of proteins without affecting any internal residue. It also enables protein selectivity by distinguishing *N*-Gly from all the other N-terminal amino acids in a complex mixture of proteins. In another report, the Gly tag technology inspired azido pyridoxal derivatives that delivered residue-specific azidolation.^118^ Engaging a unique combination of functionalities for an irreversible transformation can offer new gateways for precisely engineered bioconjugates. It would be interesting to see if chemical transformations can harness a *C*^α^-H p*K*_a_ of another unique residue. Besides, the side chain residues can generate a proximal electrophilic system to engage the *C*^α^-H-enabled nucleophilic intermediate. One can also imagine a side-chain residue to generate an intermediate that can serve as a leaving group in cooperation with *C*^α^-H abstraction. Such a case could lead to the formation of chemically orthogonal dehydroalanine. If extended to an internal residue, these approaches will likely encounter multiple solvent-accessible copies. Hence, the question of N-residue specificity will be redefined to site selectivity.

## Conclusion

Protein bioconjugates have established their immense value at the chemistry–biology–medicine interface. However, chemical methods enabling control over precision are vital to meet the technological demands. This makes it essential to understand the bottlenecks in the development of new routes for selectively modifying proteins. The functional group ecosystem of a protein is complex, and the interplay of multiple operating parameters adds to the challenge. That is why hit-and-trial or directed screening has been a preferred option for developing a new method.

The DIN theory, in this Outlook, argues that the selectivity attributes can be disintegrated through a multistep chemical transformation. In turn, it could enable the exploration of new reactivity dimensions, better prediction of potential products, and hypothesis-driven research. We have outlined three representative routes (b–d) to exemplify how such deconvolutions led to additional selectivity or specificity attributes. It would be interesting to see how new dimensions add to this repertoire with time. The methods addressing all the selectivity attributes in a single step often fall short of site selectivity. Besides, their flexibility and translation for protein selectivity in complex systems are limited. Additionally, the precision in such examples displays a very high sensitivity toward the reaction parameters. The disintegration of chemoselectivity and site selectivity in two intermolecular steps renders a unique reactivity landscape and hotspots for single-site modification. Shifting the latter to an intramolecular step also opens the platform to regulating modularity. Further, the multistep deconvolution could deliver the simultaneous regulation of chemoselectivity, residue specificity, and protein selectivity. Such processes also offer access to a broader reaction parameter window without compromising the overall selectivity.

In the coming years, the DIN theory could encourage the examination of multiple unique reactive intermediates through reversible transformations. In turn, we will have access to distinct reactivity orders leading to unique bioconjugation sites. These findings will likely extend the reach of single-site bioconjugation to low-reactivity residues. It will also be interesting to see if such deconvolutions can empower high-energy intermediates for site-selective modification, contrary to their behavior. We can expect it to accelerate the field’s growth while contributing to the rapidly growing segment of biologics such as ADCs, AFCs, and conjugate vaccines. Besides, such regulations can expedite the discovery of molecules, such as covalent inhibitors, for the selective targeting of a protein.
